# Supplementary material for: A comparison of approaches to improve worst-case predictive model performance over patient subpopulations
Source: Sci Rep. 2022 Feb 28;12:3254. doi: 10.1038/s41598-022-07167-7 (PMC8885701; doi:10.1038/s41598-022-07167-7)
Supplement: Supplementary file 1 — Supplementary Information. [file 41598_2022_7167_MOESM1_ESM.pdf]

A comparison of approaches to improve worst-case predictive model performance over patient subpopulations

Stephen R. Pfohl[*,1], Haoran Zhang[2], Yizhe Xu[1],
Agata Foryciarz[1,3], Marzyeh Ghassemi[4,5], Nigam H. Shah[1]

[1]Stanford Center for Biomedical Informatics Research, Stanford University, Stanford, California 94305, USA
[2]Department of Computer Science, University of Toronto, Toronto, Ontario, Canada
[3]Department of Computer Science, Stanford University, Stanford, California 94305, USA
[4]Department of Electrical Engineering and Computer Science, Massachusetts Institute of Technology, Cambridge, Massachusetts 02139, USA
[5]Institute for Medical and Evaluative Sciences, Massachusetts Institute of Technology, Cambridge, Massachusetts 02139, USA
*Correspondence to: spfohl@stanford.edu

A Supplementary Tables

Table A1: Characteristics for cohorts drawn from MIMIC-III and the eICU Collaborative Research Database to predict in-hospital mortality 48 hours after ICU admission, following Harutyunyan et al. [39] and Sheikhalishahi et al. [40]. Data are grouped based on age, sex, and the race and ethnicity category. Shown, for each group, is the number of patients extracted and the incidence of in-hospital mortality.

	MIMIC-III [39]		eICU [40]	
Group	Count	In-hospital mortality	Count	In-hospital mortality
[18-30)	873	0.056	1,301	0.073
[30-45)	1,890	0.086	2,578	0.074
[45-55)	2,916	0.097	4,038	0.090
[55-65)	4,047	0.109	6,458	0.105
[65-75)	4,410	0.130	7,311	0.116
[75-90)	7,003	0.184	8,994	0.150
Female	9,510	0.135	13,929	0.116
Male	11,629	0.130	16,751	0.114
Black or African American	2,015	0.092	3,402	0.096
Other race/ethnicity	4,129	0.163	3,623	0.114
White	14,995	0.129	23,655	0.118

Table A2: Disaggregated cohort characteristics for patients drawn from STARR included in the “Other race/ethnicity” group. Shown, for each group, is the number of patients extracted and the incidence of hospital mortality, prolonged length of stay, and 30-day readmission.

		Outcome Incidence		
Group	Count	Mortality	Prolonged LOS	30-day Readmission
American Indian or Alaska Native	502	0.0259	0.227	0.0558
Native Hawaiian and Pacific Islander	2,407	0.0237	0.210	0.0656
Other or no matching race/ethnicity	14,789	0.0226	0.200	0.0449
Patient declined or refused to state	1,587	0.00945	0.121	0.0246
Unknown race/ethnicity	5,348	0.0570	0.240	0.0323

Table A3: Disaggregated cohort characteristics for MIMIC-III and the eICU Collaborative Research Database to predict in-hospital mortality 48 hours after ICU admission that were included in the “Other race/ethnicity” group. Shown, for each group, is the number of patients extracted and the incidence of in-hospital mortality.

	MIMIC-III [39]		eICU [40]	
Group	Count	In-hospital mortality	Count	In-hospital mortality
Asian	492	0.138	492	0.118
Hispanic or Latino	679	0.0810	1,111	0.113
Other or unknown race/ethnicity			1,659	0.118
Other or no matching race/ethnicity	573	0.140		
Patient refused or declined to state	199	0.126		
Unknown race/ethnicity	2,186	0.203		

B Supplementary Figures

In this section, we provide figures containing the results for each of the prediction tasks evaluated. Supplementary Figures B1 and B2 contain overlapping results with Figures 2 and 3 presented in main text. The results for the models learned from the inpatient admission cohort derived from STARR are presented in Section B.1. Section B.2 contains the results for models derived from the MIMIC-III and eICU databases.

B.1 Results for models that predict in-hospital mortality, prolonged length of stay, and 30-day readmission from STARR

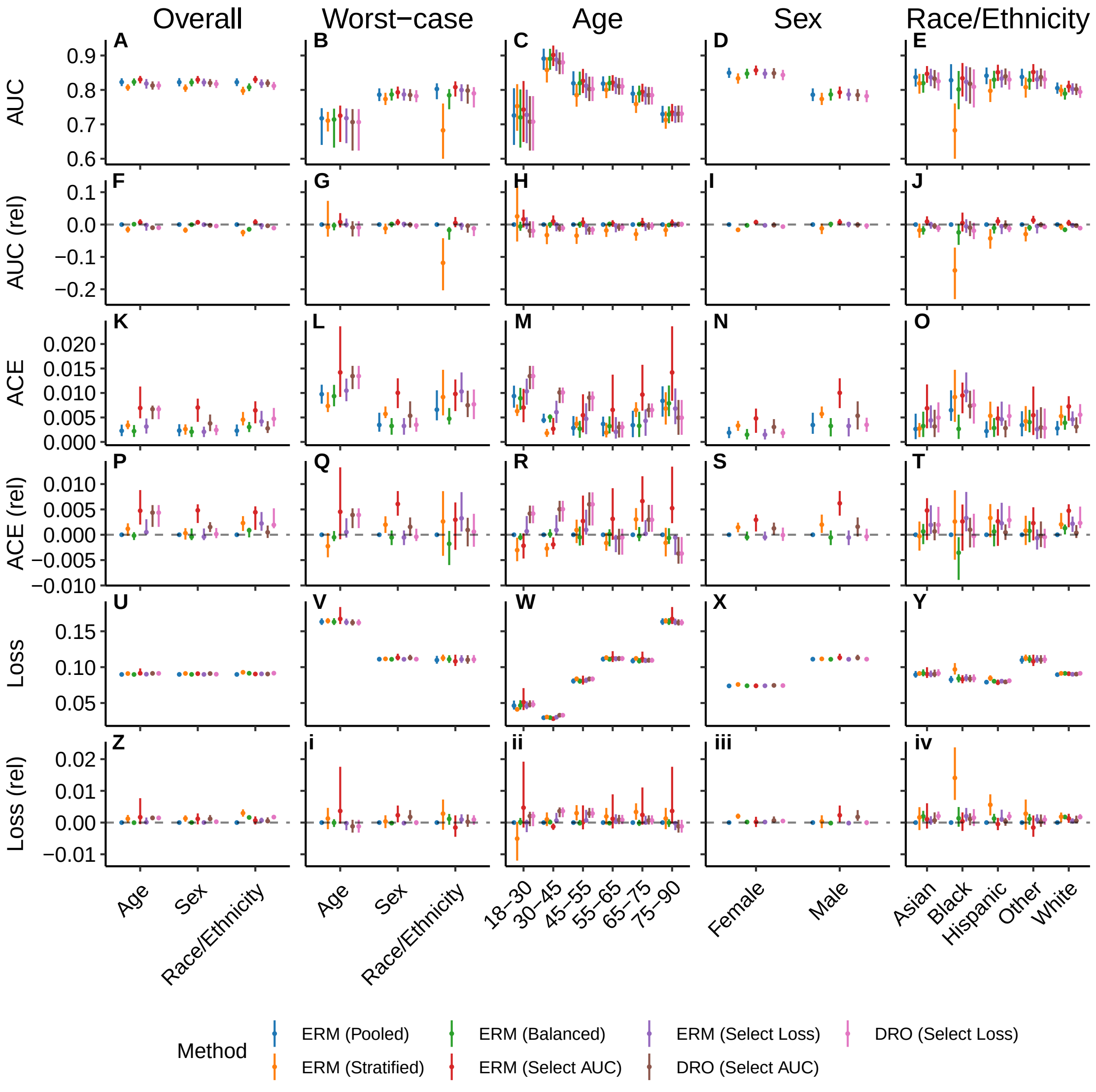

Supplementary Figure B1: The performance of models that to predict in-hospital mortality at admission using data derived from the STARR database. Results shown are the area under the receiver operating characteristic curve (AUC), absolute calibration error (ACE), and the loss assessed in the overall population, on each subpopulation, and in the worst-case over subpopulations for models trained with pooled, stratified, and balanced empirical risk minimization (ERM) and a range of distributionally robust optimization (DRO) training objectives. For both pooled ERM and DRO, we show the models selected based on worst-case model selection criteria that perform selection based on the worst-case subpopulation AUC (Select AUC) or loss (Select Loss). Model selection occurs over all relevant training objectives, sampling rules, and early-stopping criteria. Error bars indicate absolute and relative 95% confidence intervals derived with the percentile bootstrap with 1,000 iterations. Relative performance (suffixed by "rel") is assessed with respect to the performance of models derived with ERM applied to the entire training dataset.

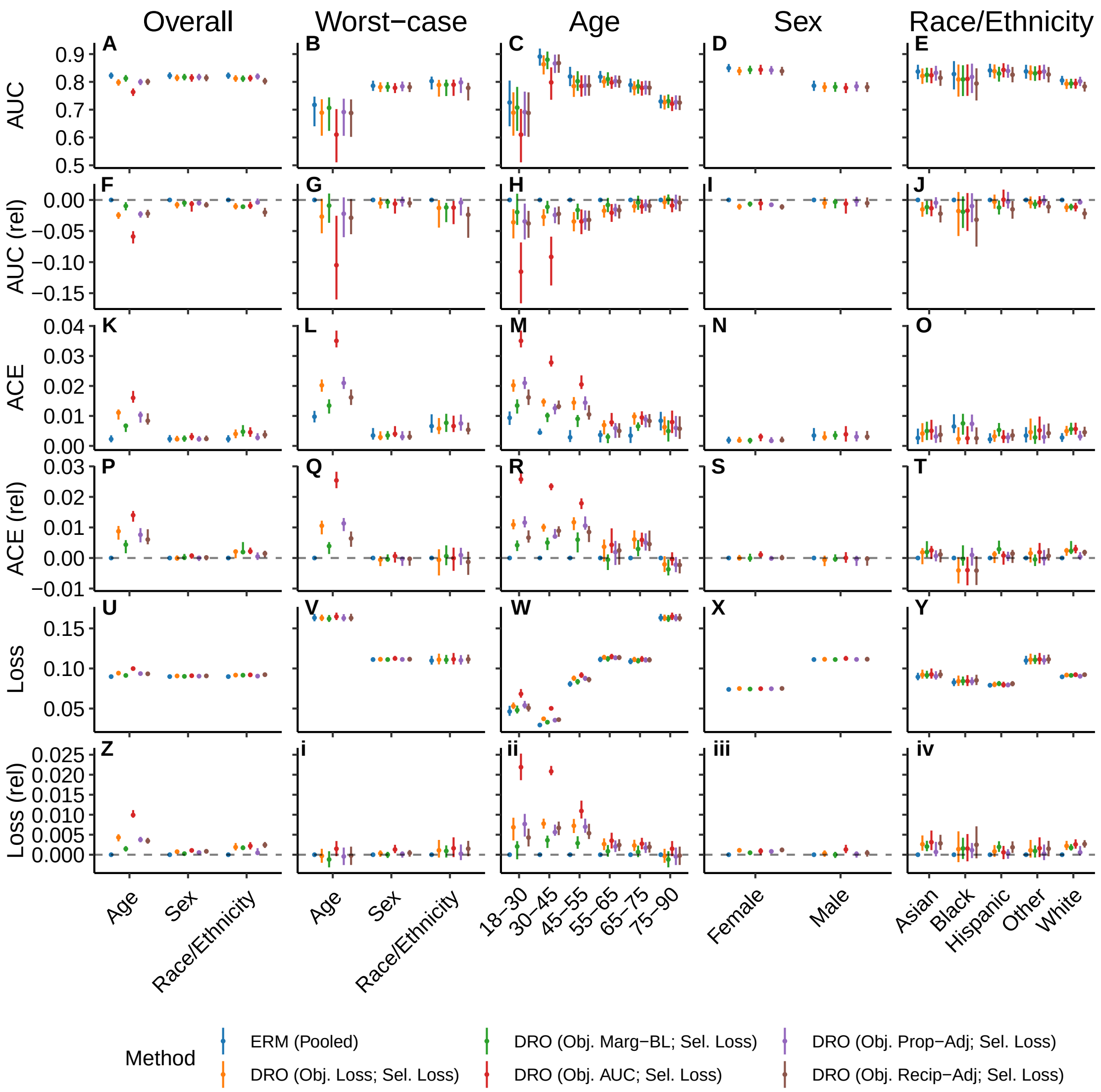

Supplementary Figure B2: The performance of models trained with distributionally robust optimization (DRO) training objectives to predict in-hospital mortality at admission using data derived from the STARR database, following model selection based on worst-case loss over subpopulations. Results shown are the area under the receiver operating characteristic curve (AUC), absolute calibration error (ACE), and the loss assessed in the overall population, on each subpopulation, and in the worst-case over subpopulations for models trained with the unadjusted DRO training objective (Obj. Loss), the adjusted training objective that subtracts the marginal entropy in the outcome (Obj. Marg-BL), the training objective that uses the AUC-based update (Obj. AUC), and training objectives that use adjustments that scale proportionally (Obj. Prop-Adj) and inversely to the size of the group (Obj. Recip-Adj). Error bars indicate absolute and relative 95% confidence intervals derived with the percentile bootstrap with 1,000 iterations. Relative performance (suffixed by “rel”) is assessed with respect to the performance of models derived with ERM applied to the entire training dataset.

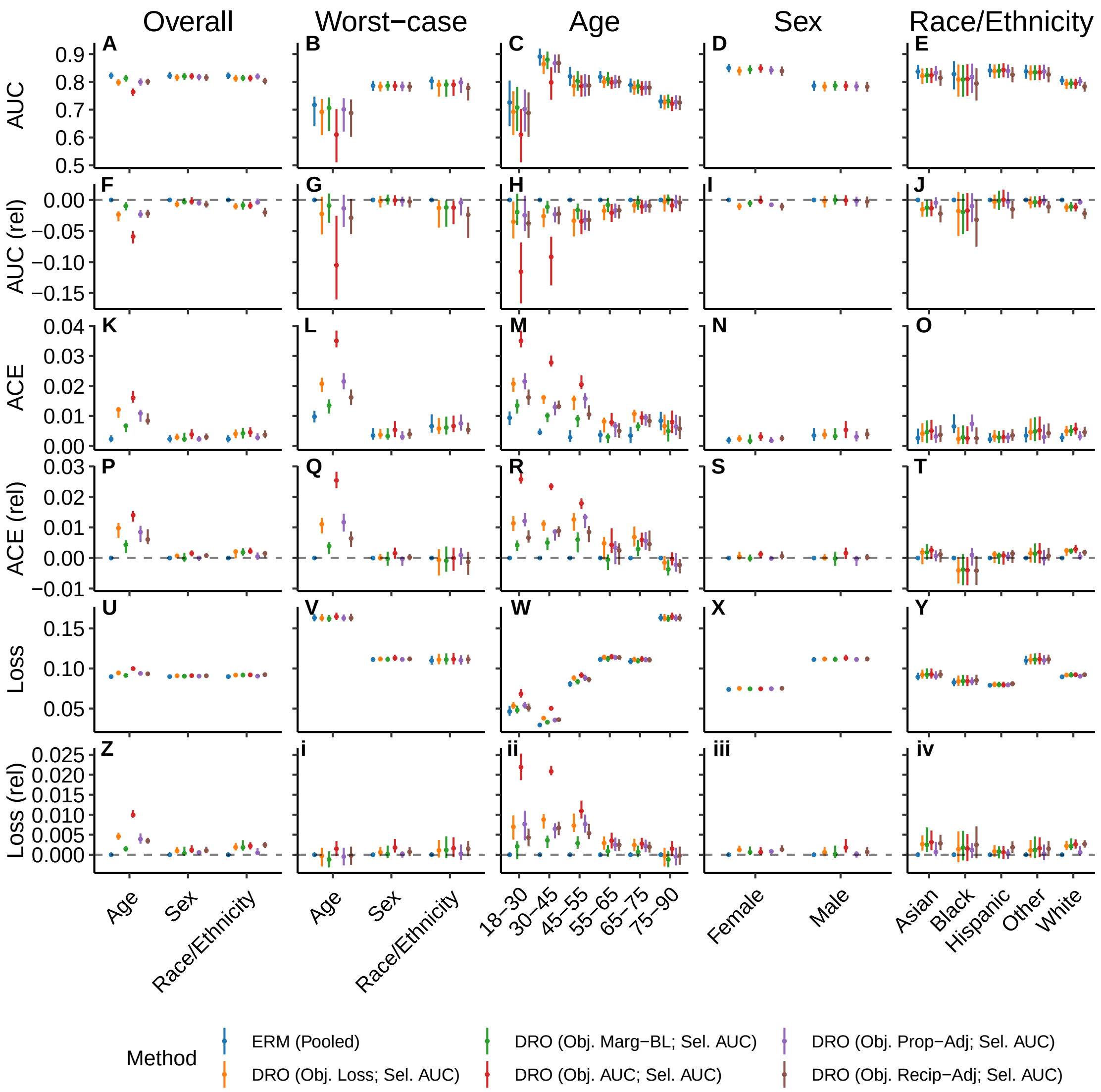

Supplementary Figure B3: The performance of models trained with distributionally robust optimization (DRO) training objectives to predict in-hospital mortality at admission using data derived from the STARR database, following model selection based on worst-case AUC over subpopulations. Results shown are the area under the receiver operating characteristic curve (AUC), absolute calibration error (ACE), and the loss assessed in the overall population, on each subpopulation, and in the worst-case over subpopulations for models trained with the unadjusted DRO training objective (Obj. Loss), the adjusted training objective that subtracts the marginal entropy in the outcome (Obj. Marg-BL), the training objective that uses the AUC-based update (Obj. AUC), and training objectives that use adjustments that scale proportionally (Obj. Prop-Adj) and inversely to the size of the group (Obj. Recip-Adj). Error bars indicate absolute and relative 95% confidence intervals derived with the percentile bootstrap with 1,000 iterations. Relative performance (suffixed by "rel") is assessed with respect to the performance of models derived with ERM applied to the entire training dataset.

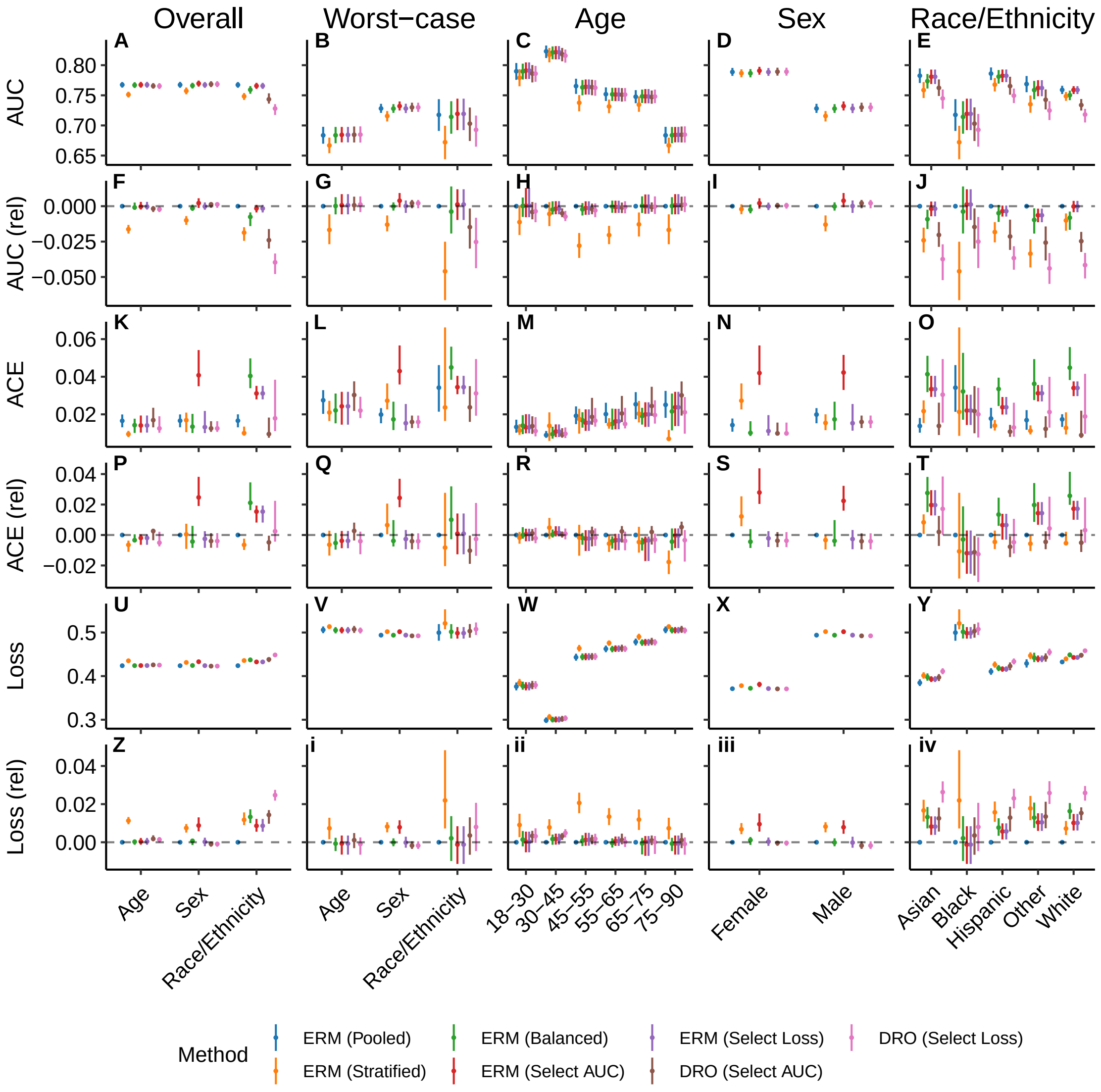

Supplementary Figure B4: The performance of models that predict prolonged length of stay at admission using data derived from the STARR database. Results shown are the area under the receiver operating characteristic curve (AUC), absolute calibration error (ACE), and the loss assessed in the overall population, on each subpopulation, and in the worst-case over subpopulations for models trained with pooled, stratified, and balanced empirical risk minimization (ERM) and a range of distributionally robust optimization (DRO) training objectives. For both pooled ERM and DRO, we show the models selected based on worst-case model selection criteria that perform selection based on the worst-case subpopulation AUC (Select AUC) or loss (Select Loss). Model selection occurs over all relevant training objectives, sampling rules, and early-stopping criteria. Error bars indicate absolute and relative 95% confidence intervals derived with the percentile bootstrap with 1,000 iterations. Relative performance (suffixed by "rel") is assessed with respect to the performance of models derived with ERM applied to the entire training dataset.

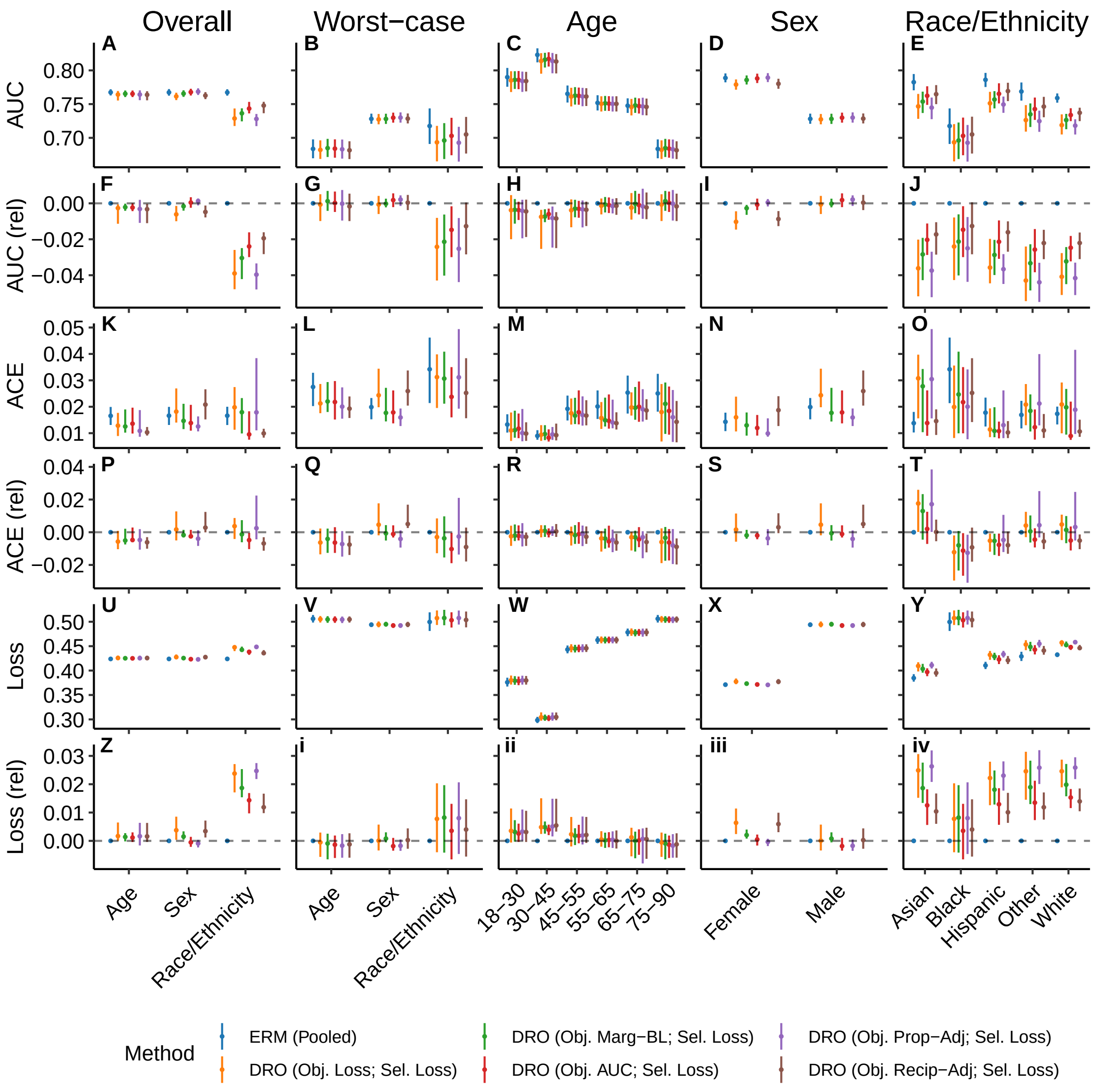

Supplementary Figure B5: The performance of models trained with distributionally robust optimization (DRO) training objectives to predict prolonged length of stay at admission using data derived from the STARR database, following model selection based on worst-case loss over subpopulations. Results shown are the area under the receiver operating characteristic curve (AUC), absolute calibration error (ACE), and the loss assessed in the overall population, on each subpopulation, and in the worst-case over subpopulations for models trained with the unadjusted DRO training objective (Obj. Loss), the adjusted training objective that subtracts the marginal entropy in the outcome (Obj. Marg-BL), the training objective that uses the AUC-based update (Obj. AUC), and training objectives that use adjustments that scale proportionally (Obj. Prop-Adj) and inversely to the size of the group (Obj. Recip-Adj). Error bars indicate absolute and relative 95% confidence intervals derived with the percentile bootstrap with 1,000 iterations. Relative performance (suffixed by "rel") is assessed with respect to the performance of models derived with ERM applied to the entire training dataset.

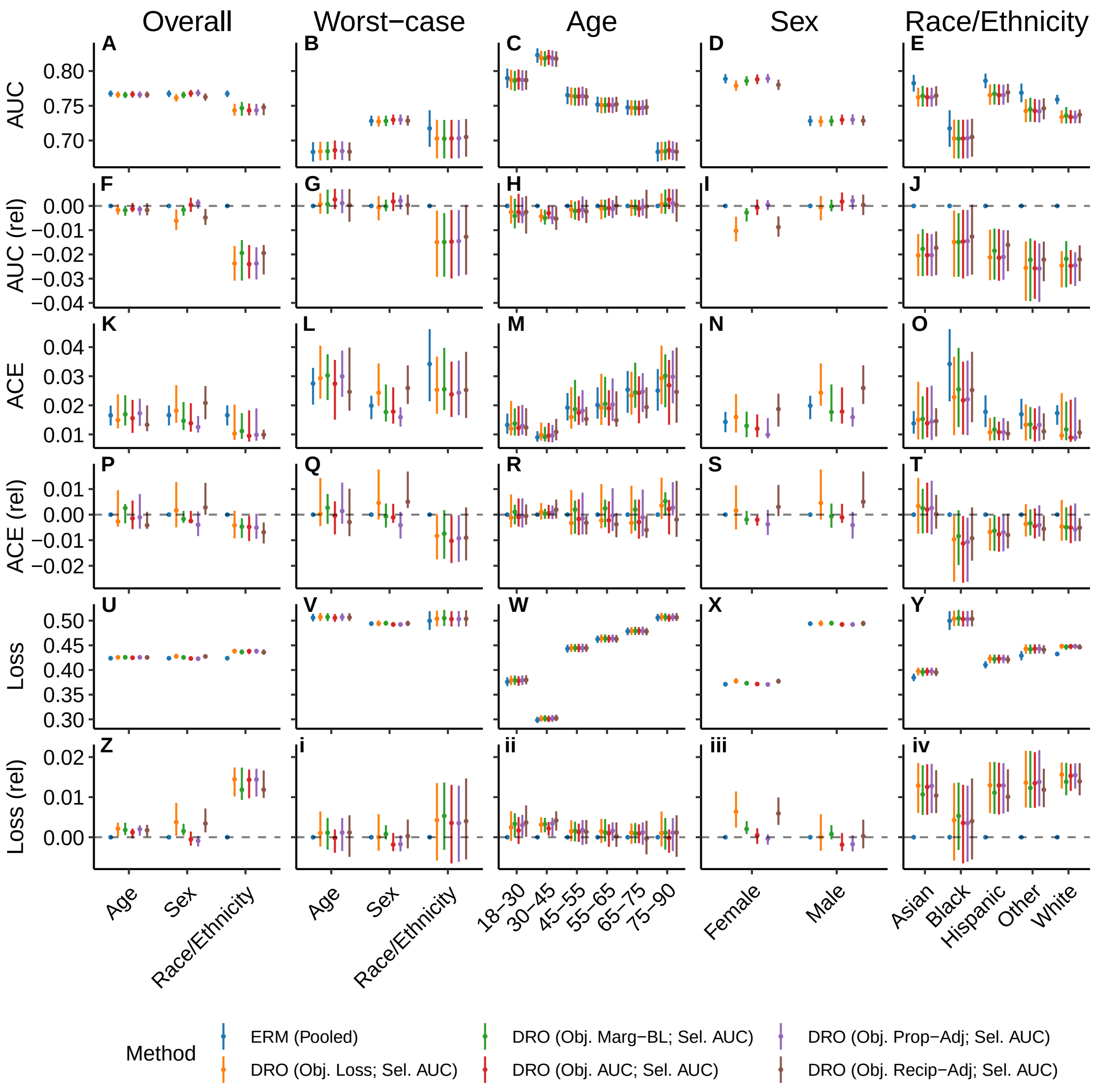

Supplementary Figure B6: The performance of models trained with distributionally robust optimization (DRO) training objectives to predict prolonged length of stay at admission using data derived from the STARR database, following model selection based on worst-case AUC over subpopulations. Results shown are the area under the receiver operating characteristic curve (AUC), absolute calibration error (ACE), and the loss assessed in the overall population, on each subpopulation, and in the worst-case over subpopulations for models trained with the unadjusted DRO training objective (Obj. Loss), the adjusted training objective that subtracts the marginal entropy in the outcome (Obj. Marg-BL), the training objective that uses the AUC-based update (Obj. AUC), and training objectives that use adjustments that scale proportionally (Obj. Prop-Adj) and inversely to the size of the group (Obj. Recip-Adj). Error bars indicate absolute and relative 95% confidence intervals derived with the percentile bootstrap with 1,000 iterations. Relative performance (suffixed by "rel") is assessed with respect to the performance of models derived with ERM applied to the entire training dataset.

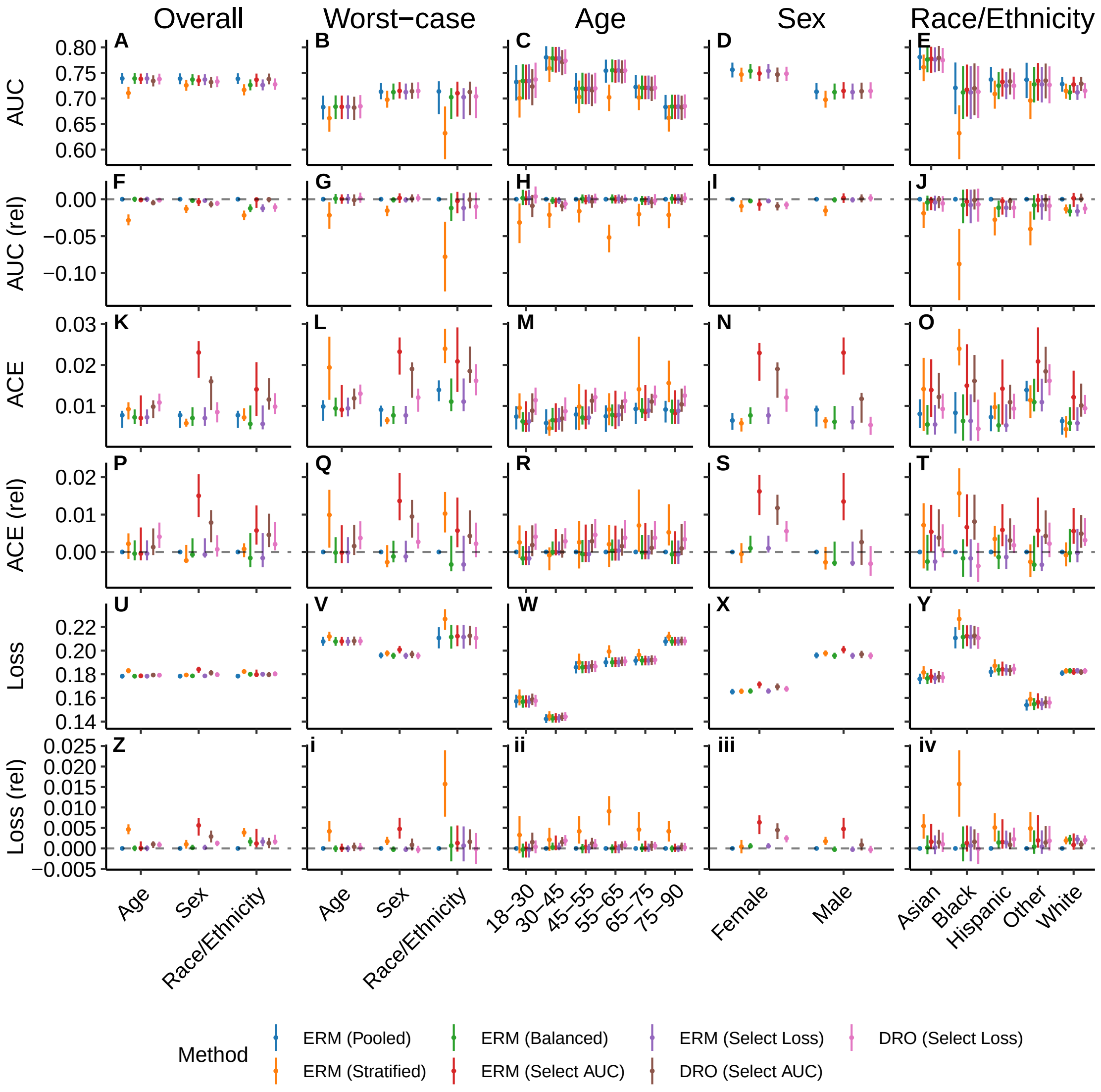

Supplementary Figure B7: The performance of models that predict 30-day readmission at admission using data derived from the STARR database. Results shown are the area under the receiver operating characteristic curve (AUC), absolute calibration error (ACE), and the loss assessed in the overall population, on each subpopulation, and in the worst-case over subpopulations for models trained with pooled, stratified, and balanced empirical risk minimization (ERM) and a range of distributionally robust optimization (DRO) training objectives. For both pooled ERM and DRO, we show the models selected based on worst-case model selection criteria that perform selection based on the worst-case subpopulation AUC (Select AUC) or loss (Select Loss). Model selection occurs over all relevant training objectives, sampling rules, and early-stopping criteria. Error bars indicate absolute and relative 95% confidence intervals derived with the percentile bootstrap with 1,000 iterations. Relative performance (suffixed by "rel") is assessed with respect to the performance of models derived with ERM applied to the entire training dataset.

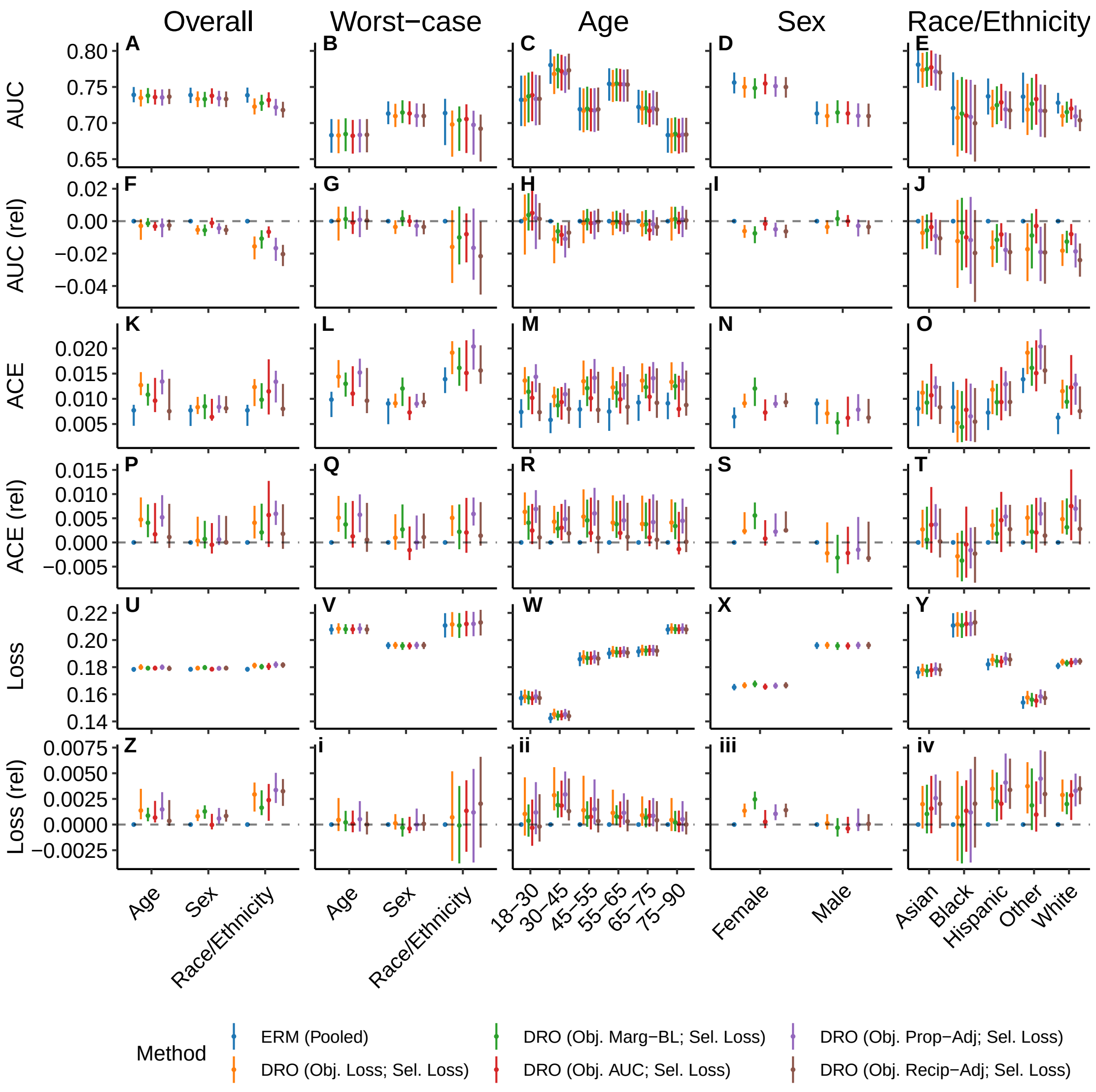

Supplementary Figure B8: The performance of models trained with distributionally robust optimization (DRO) training objectives to predict 30-day readmission at admission using data derived from the STARR database, following model selection based on worst-case loss over subpopulations Results shown are the area under the receiver operating characteristic curve (AUC), absolute calibration error (ACE), and the loss assessed in the overall population, on each subpopulation, and in the worst-case over subpopulations for models trained with the unadjusted DRO training objective (Obj. Loss), the adjusted training objective that subtracts the marginal entropy in the outcome (Obj. Marg-BL), the training objective that uses the AUC-based update (Obj. AUC), and training objectives that use adjustments that scale proportionally (Obj. Prop-Adj) and inversely to the size of the group (Obj. Recip-Adj). Error bars indicate absolute and relative 95% confidence intervals derived with the percentile bootstrap with 1,000 iterations. Relative performance (suffixed by "rel") is assessed with respect to the performance of models derived with ERM applied to the entire training dataset.

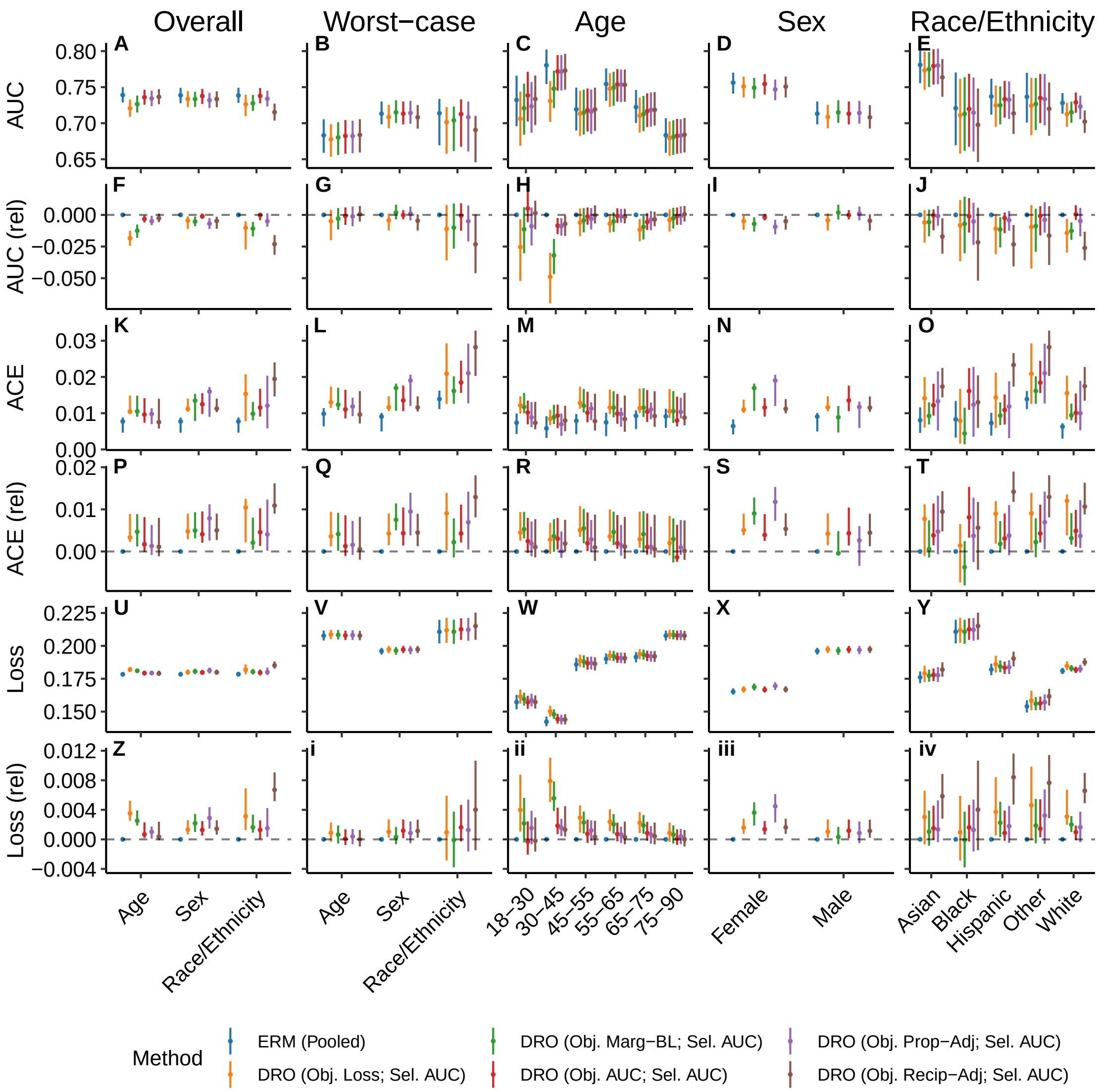

Supplementary Figure B9: The performance of models trained with distributionally robust optimization (DRO) training objectives to predict 30-day readmission at admission using data derived from the STARR database, following model selection based on worst-case AUC over subpopulations. Results shown are the area under the receiver operating characteristic curve (AUC), absolute calibration error (ACE), and the loss assessed in the overall population, on each subpopulation, and in the worst-case over subpopulations for models trained with the unadjusted DRO training objective (Obj. Loss), the adjusted training objective that subtracts the marginal entropy in the outcome (Obj. Marg-BL), the training objective that uses the AUC-based update (Obj. AUC), and training objectives that use adjustments that scale proportionally (Obj. Prop-Adj) and inversely to the size of the group (Obj. Recip-Adj). Error bars indicate absolute and relative 95% confidence intervals derived with the percentile bootstrap with 1,000 iterations. Relative performance (suffixed by "rel") is assessed with respect to the performance of models derived with ERM applied to the entire training dataset.

B.2 Results for models that predict in-hospital mortality from intensive care databases

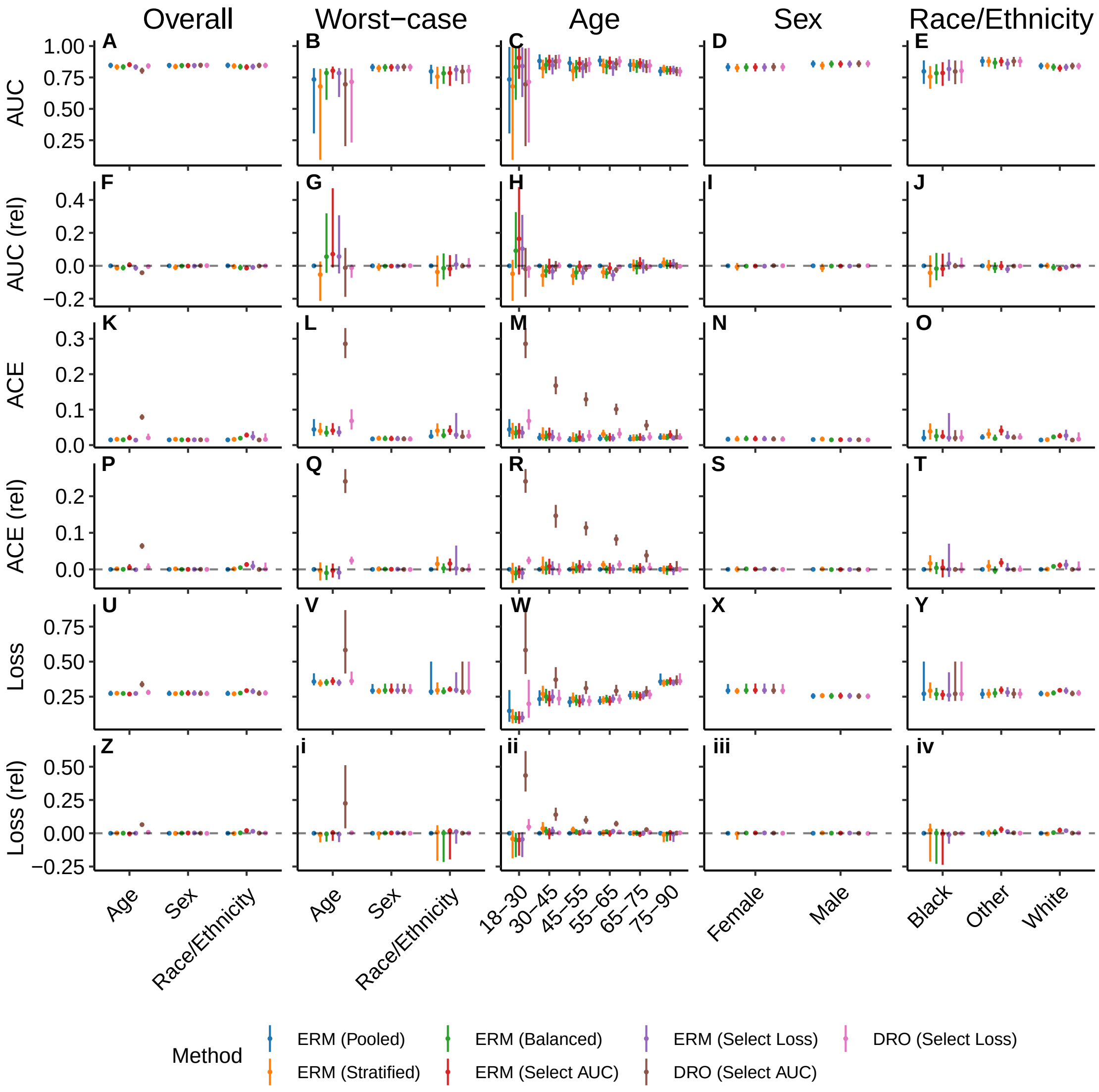

Supplementary Figure B10: The performance of models that predict in-hospital mortality using features derived from data recorded in the first 48 hours of a patient's ICU stay for data derived from the MIMIC-III database, following Harutyunyan et al. [39]. Results shown are the area under the receiver operating characteristic curve (AUC), absolute calibration error (ACE), and the loss assessed in the overall population, on each subpopulation, and in the worst-case over subpopulations for models trained with pooled, stratified, and balanced empirical risk minimization (ERM) and a range of distributionally robust optimization (DRO) training objectives. For both pooled ERM and DRO, we show the models selected based on worst-case model selection criteria that perform selection based on the worst-case subpopulation AUC (Select AUC) or loss (Select Loss). Model selection occurs over all relevant training objectives, sampling rules, and early-stopping criteria. Error bars indicate absolute and relative 95% confidence intervals derived with the percentile bootstrap with 1,000 iterations. Relative performance (suffixed by "rel") is assessed with respect to the performance of models derived with ERM applied to the entire training dataset.

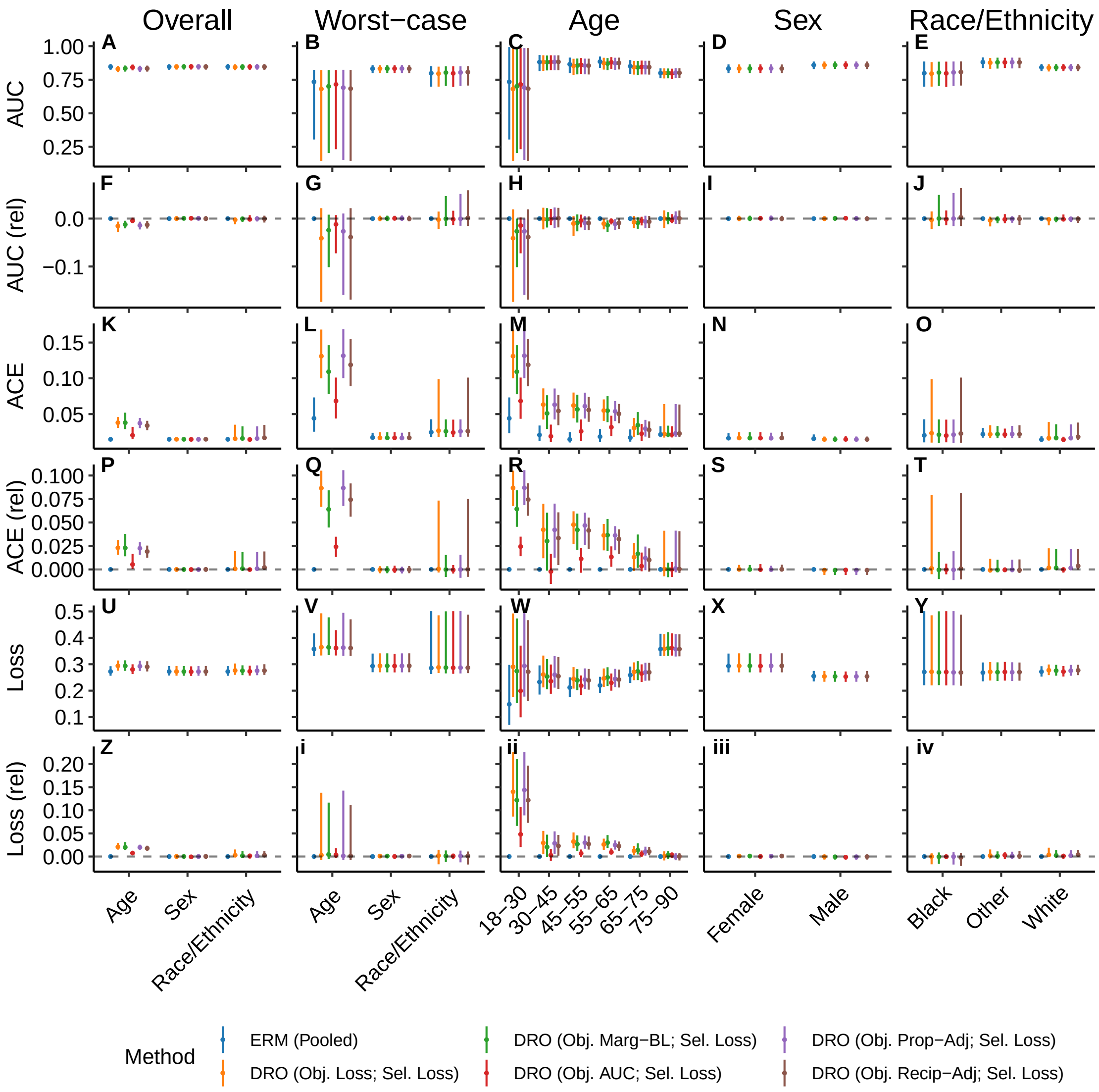

Supplementary Figure B11: The performance of models trained with distributionally robust optimization (DRO) training objectives to predict in-hospital mortality using features extracted from data derived from the first 48 hours of a patient's ICU stay using data derived from the MIMIC-III database, following Harutyunyan et al. [39], following model selection based on worst-case loss over subpopulations Results shown are the area under the receiver operating characteristic curve (AUC), absolute calibration error (ACE), and the loss assessed in the overall population, on each subpopulation, and in the worst-case over subpopulations for models trained with the unadjusted DRO training objective (Obj. Loss), the adjusted training objective that subtracts the marginal entropy in the outcome (Obj. Marg-BL), the training objective that uses the AUC-based update (Obj. AUC), and training objectives that use adjustments that scale proportionally (Obj. Prop-Adj) and inversely to the size of the group (Obj. Recip-Adj). Error bars indicate absolute and relative 95% confidence intervals derived with the percentile bootstrap with 1,000 iterations. Relative performance (suffixed by "rel") is assessed with respect to the performance of models derived with ERM applied to the entire training dataset.

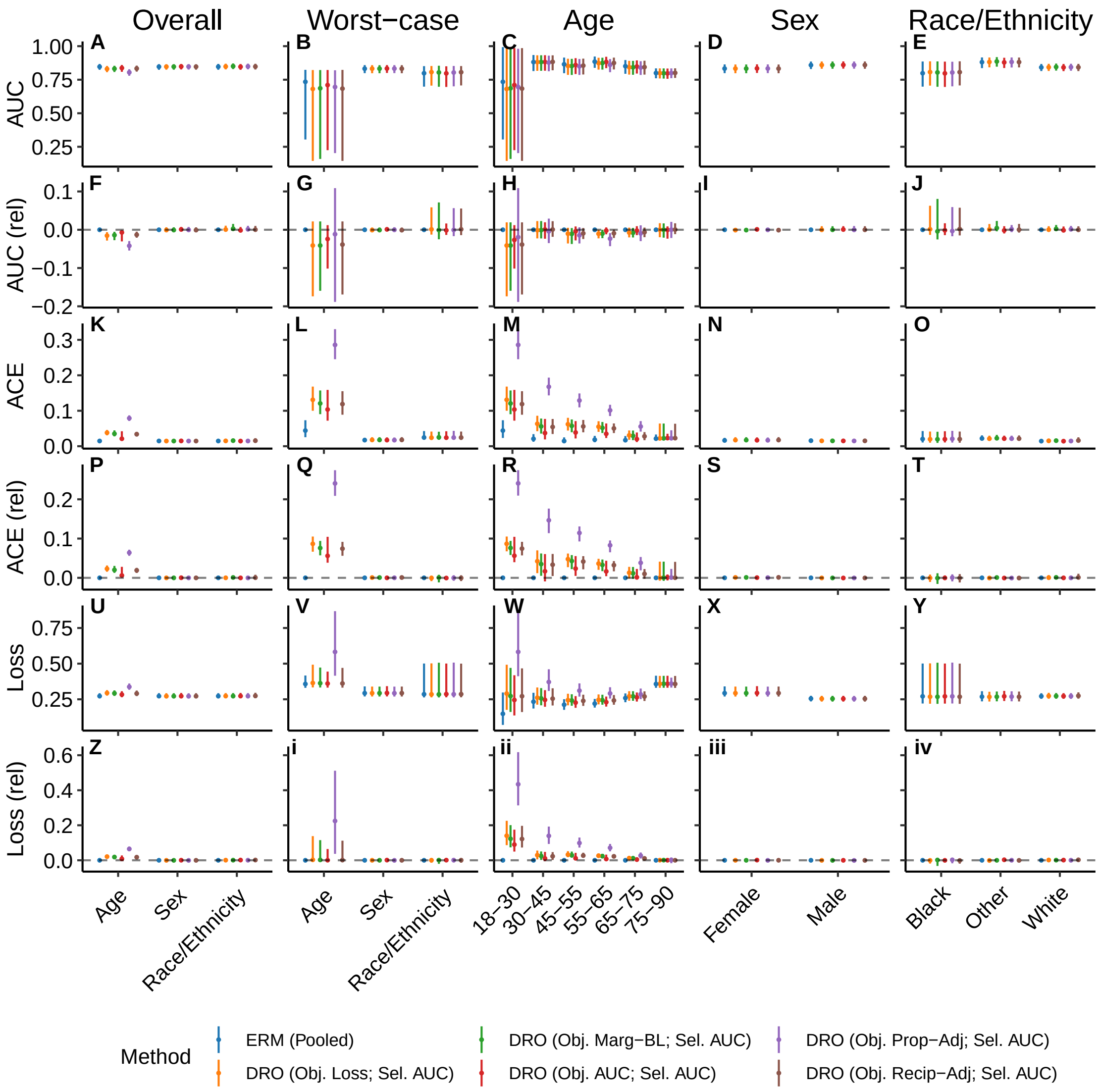

Supplementary Figure B12: The performance of models trained with distributionally robust optimization (DRO) training objectives to predict in-hospital mortality using features extracted from data derived from the first 48 hours of a patient's ICU stay using data derived from the MIMIC-III database, following Harutyunyan et al. [39], following model selection based on worst-case AUC over subpopulations. Results shown are the area under the receiver operating characteristic curve (AUC), absolute calibration error (ACE), and the loss assessed in the overall population, on each subpopulation, and in the worst-case over subpopulations for models trained with the unadjusted DRO training objective (Obj. Loss), the adjusted training objective that subtracts the marginal entropy in the outcome (Obj. Marg-BL), the training objective that uses the AUC-based update (Obj. AUC), and training objectives that use adjustments that scale proportionally (Obj. Prop-Adj) and inversely to the size of the group (Obj. Recip-Adj). Error bars indicate absolute and relative 95% confidence intervals derived with the percentile bootstrap with 1,000 iterations. Relative performance (suffixed by "rel") is assessed with respect to the performance of models derived with ERM applied to the entire training dataset.

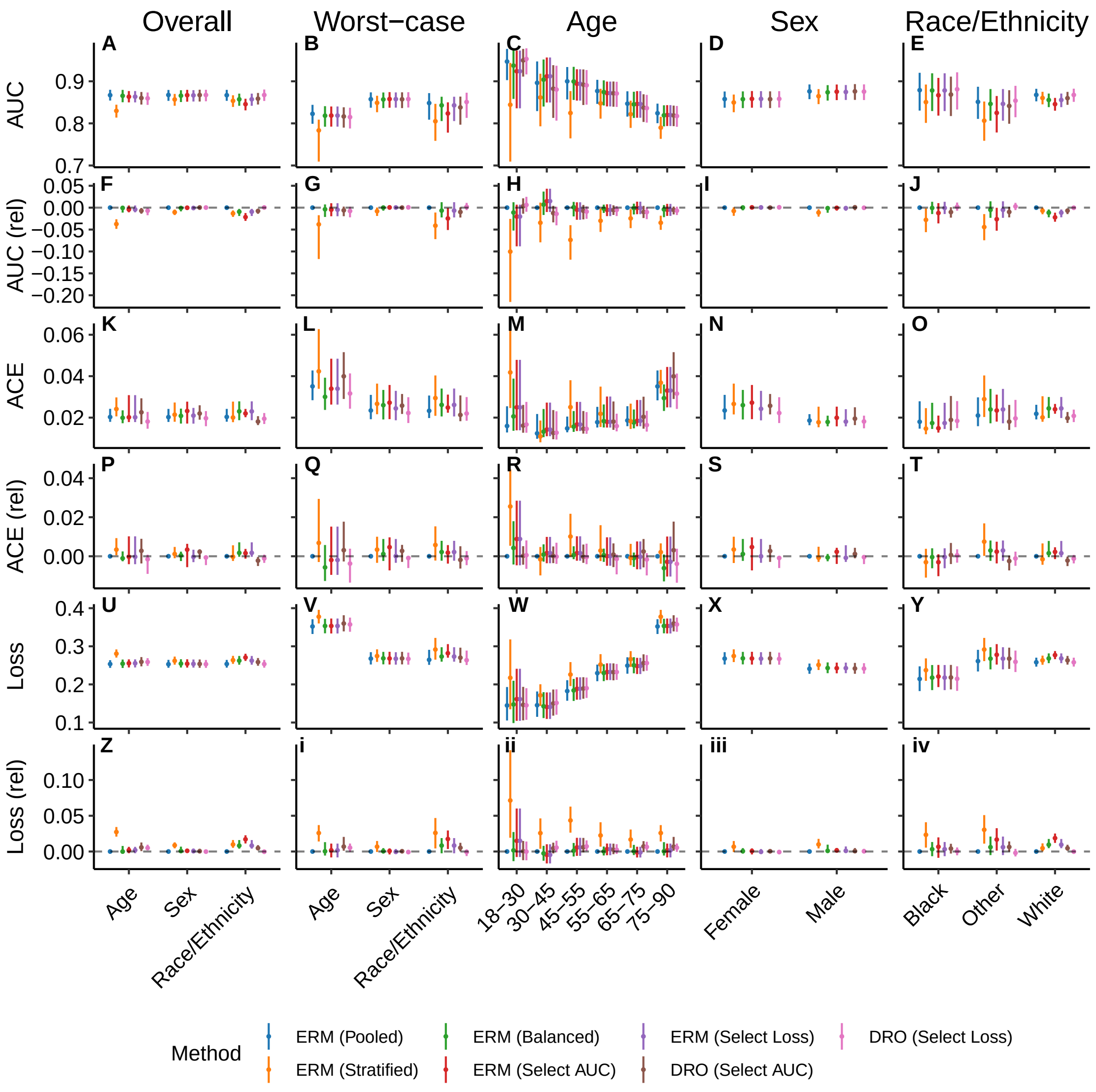

Supplementary Figure B13: The performance of models that predict in-hospital mortality using features derived from data recorded in the first 48 hours of a patient's ICU stay for data derived from the eICU database, following Sheikhalishahi et al. [40]. Results shown are the area under the receiver operating characteristic curve (AUC), absolute calibration error (ACE), and the loss assessed in the overall population, on each subpopulation, and in the worst-case over subpopulations for models trained with pooled, stratified, and balanced empirical risk minimization (ERM) and a range of distributionally robust optimization (DRO) training objectives. For both pooled ERM and DRO, we show the models selected based on worst-case model selection criteria that perform selection based on the worst-case subpopulation AUC (Select AUC) or loss (Select Loss). Model selection occurs over all relevant training objectives, sampling rules, and early-stopping criteria. Error bars indicate absolute and relative 95% confidence intervals derived with the percentile bootstrap with 1,000 iterations. Relative performance (suffixed by "rel") is assessed with respect to the performance of models derived with ERM applied to the entire training dataset.

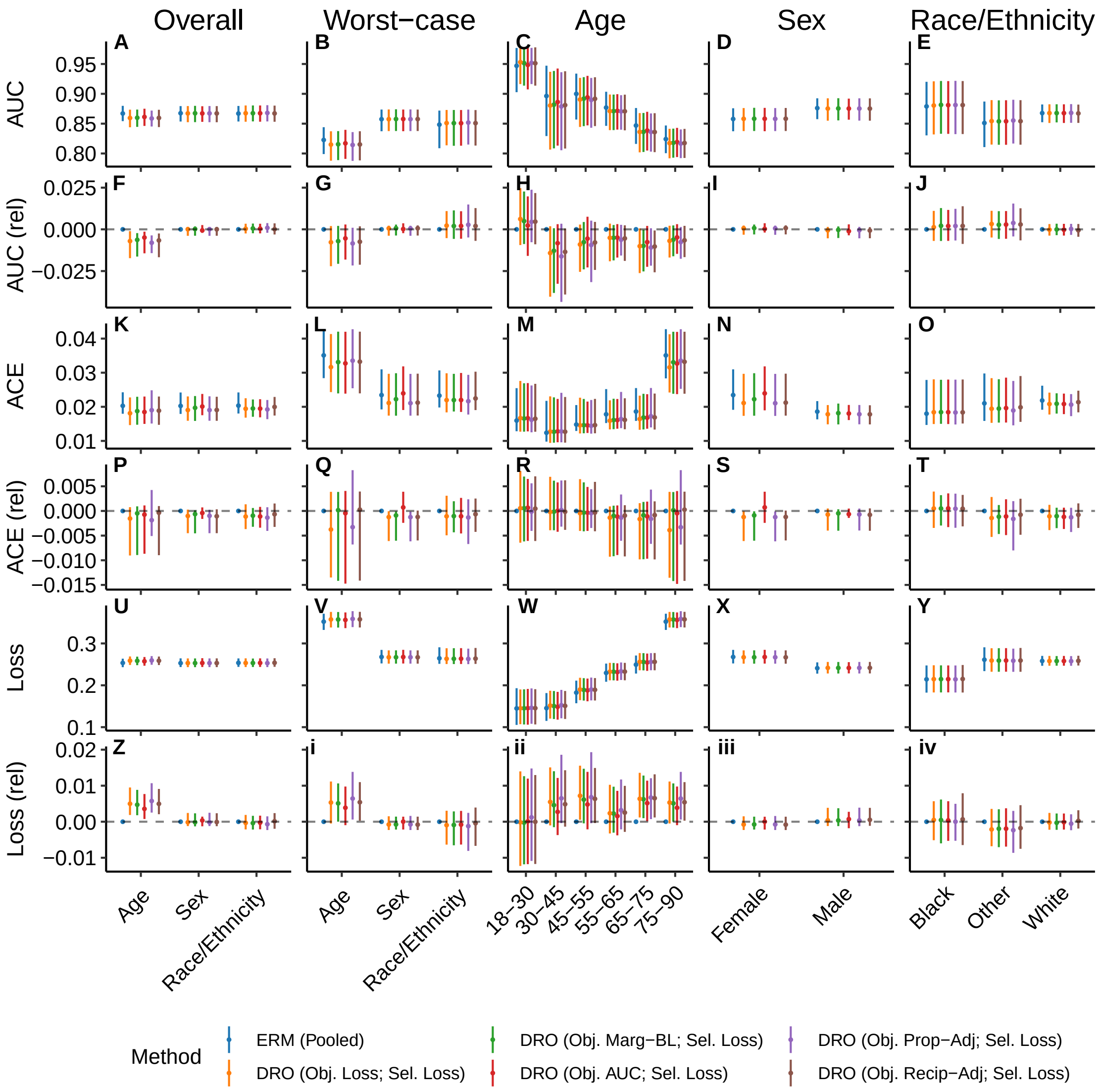

Supplementary Figure B14: The performance of models trained with distributionally robust optimization (DRO) training objectives to predict in-hospital mortality using features extracted from data derived from the first 48 hours of a patient's ICU stay using data derived from the eICU database, following Sheikhalishahi et al. [40], following model selection based on worst-case loss over subpopulations Results shown are the area under the receiver operating characteristic curve (AUC), absolute calibration error (ACE), and the loss assessed in the overall population, on each subpopulation, and in the worst-case over subpopulations for models trained with the unadjusted DRO training objective (Obj. Loss), the adjusted training objective that subtracts the marginal entropy in the outcome (Obj. Marg-BL), the training objective that uses the AUC-based update (Obj. AUC), and training objectives that use adjustments that scale proportionally (Obj. Prop-Adj) and inversely to the size of the group (Obj. Recip-Adj). Error bars indicate absolute and relative 95% confidence intervals derived with the percentile bootstrap with 1,000 iterations. Relative performance (suffixed by "rel") is assessed with respect to the performance of models derived with ERM applied to the entire training dataset.

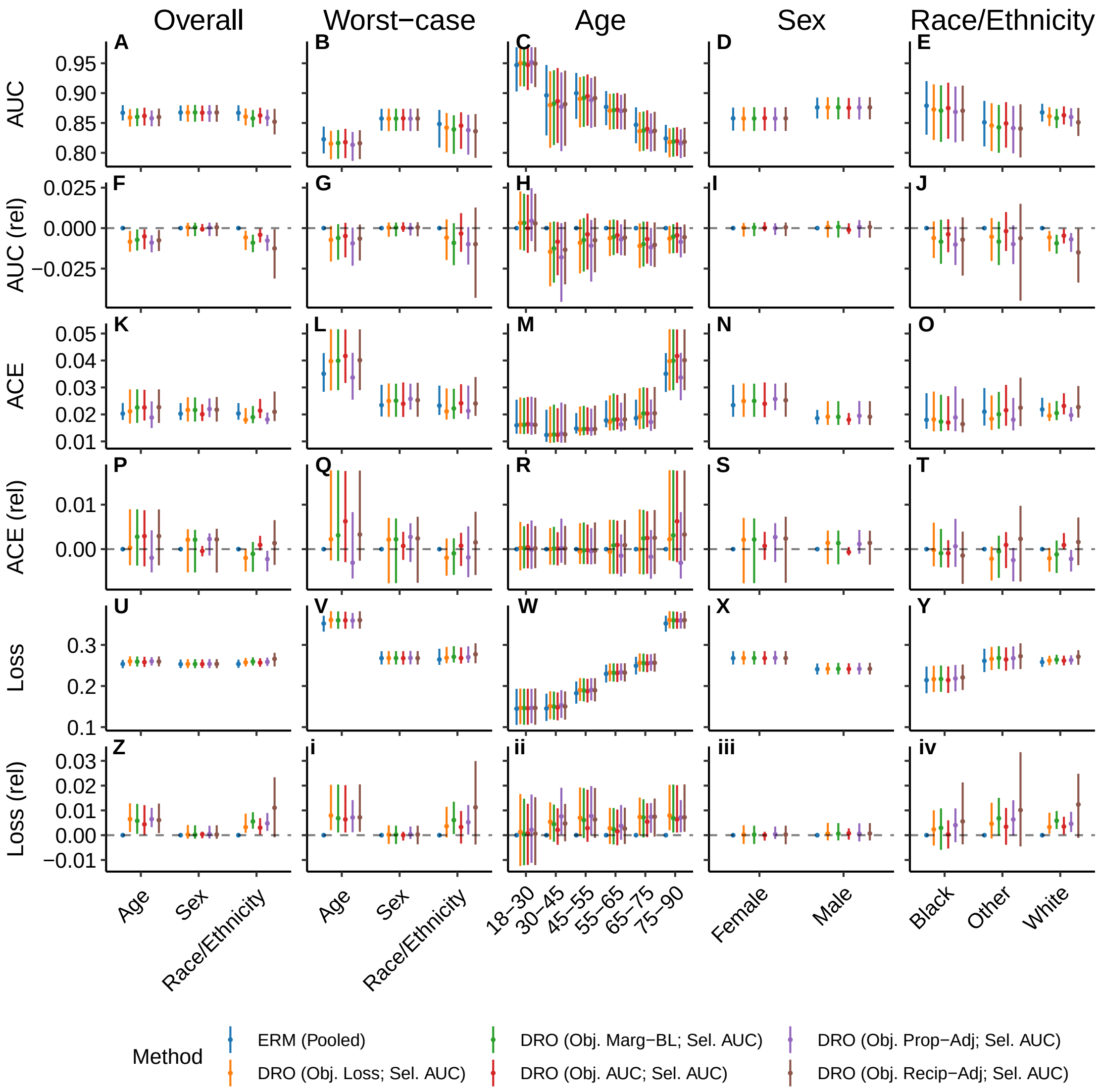

Supplementary Figure B15: The performance of models trained with distributionally robust optimization (DRO) training objectives to predict in-hospital mortality using features extracted from data derived from the first 48 hours of a patient's ICU stay using data derived from the eICU database, following Sheikhalishahi et al. [40], following model selection based on worst-case AUC over subpopulations. Results shown are the area under the receiver operating characteristic curve (AUC), absolute calibration error (ACE), and the loss assessed in the overall population, on each subpopulation, and in the worst-case over subpopulations for models trained with the unadjusted DRO training objective (Obj. Loss), the adjusted training objective that subtracts the marginal entropy in the outcome (Obj. Marg-BL), the training objective that uses the AUC-based update (Obj. AUC), and training objectives that use adjustments that scale proportionally (Obj. Prop-Adj) and inversely to the size of the group (Obj. Recip-Adj). Error bars indicate absolute and relative 95% confidence intervals derived with the percentile bootstrap with 1,000 iterations. Relative performance (suffixed by "rel") is assessed with respect to the performance of models derived with ERM applied to the entire training dataset.
